# Time-Dependent Exposures and the Fixed-Cohort Bias: Hwang et al. Respond

**DOI:** 10.1289/ehp.1103885R

**Published:** 2011-10-01

**Authors:** Bing-Fang Hwang, Yungling Leo Lee, Jouni J.K. Jaakkola

**Affiliations:** Department of Occupational Safety and Health, College of Public Health, China Medical University, Taichung, Taiwan, E-mail: bfhwang@mail.cmu.edu.tw; Institute of Epidemiology and Preventive Medicine and Research Center for Genes, Environment and Human Health, College of Public Health, National Taiwan University, Taipei, Taiwan; Center for Environmental and Respiratory Health Research, Institute of Health Sciences, University of Oulu, Oulu, Finland

Barnett expresses concerns about a potential bias in our article (Hwang et al. 2011) related to use of a fixed study period based on the date of delivery: on average a shorter duration of gestation among stillbirths compared to live births in combination with seasonal variation of exposure. We acknowledge the complexity of assessing effects of exposure with seasonal variation on the risk of stillbirth and thank Barnett for his suggestion to avoid a possible bias, which he with his colleagues illustrated through simulations of a retrospective cohort study (Strand et al. 2011). We reanalyzed the data, excluding case and control subjects following Barnett’s suggestion to quantify the “fixed cohort bias.” This led to loss of approximately 4.7% (4,480/102,575) of the subjects. The point estimates were similar with those from the original analyses, but some confidence intervals became wider (Table 1). This shows that the role of the fixed cohort bias was minimal in our study.

**Table 1:** Adjusted ORs (95% CIs) for stillbirth by average pollutant concentrations, by trimester and for the whole pregnancy (single pollutant models), following Barnett’s suggestion to address the “fixed cohort bias.”

Air pollutant	All births (gestational age > 20 weeks) Model 1a	Preterm births (gestational age < 37 weeks) Model 2b	Term births (gestational age ≥ 37 weeks) Model 3b
PM10 (10 µg/m3)						
1st trimester		1.02 (0.99–1.05)*		1.03 (1.00–1.07)		1.00 (0.96–1.04)
2nd trimester		0.97 (0.94–0.99)*		0.99 (0.95–1.03)*		0.95 (0.92–0.99)*
3rd trimester		0.97 (0.95–1.00)*		0.97 (0.92–1.02)		0.97 (0.92–1.02)*
Whole pregnancy		0.97 (0.95–1.02)*		1.01 (0.96–1.06)*		0.96 (0.91–1.01)*
SO2 (1 ppb)						
1st trimester		1.02 (1.00–1.04)		1.04 (1.01–1.06)*		1.00 (0.97–1.03)*
2nd trimester		1.00 (0.98–1.02)		1.02 (0.99–1.04)*		0.99 (0.96–1.02)
3rd trimester		1.00 (0.98–1.02)		1.01 (0.97–1.04)		1.01 (0.97–1.04)*
Whole pregnancy		1.01 (0.99–1.03)		1.03 (1.00–1.06)		0.99 (0.97–1.02)*
NO2 (10 ppb)						
1st trimester		1.01 (0.96–1.07)		1.05 (0.97–1.13)*		0.98 (0.90–1.06)
2nd trimester		0.97 (0.92–1.02)*		1.00 (0.93–1.08)*		0.95 (0.88–1.02)
3rd trimester		0.98 (0.92–1.04)		0.98 (0.89–1.08)		0.98 (0.89–1.08)*
Whole pregnancy		0.98 (0.93–1.05)*		1.02 (0.94–1.11)*		0.96 (0.88–1.05)
CO (100 ppb)						
1st trimester		1.00 (0.98–1.02)		1.00 (0.97–1.02)*		1.01 (0.98–1.04)
2nd trimester		1.00 (0.98–1.02)*		0.99 (0.96–1.01)*		1.01 (0.98–1.03)
3rd trimester		1.01 (0.99–1.03)*		0.98 (0.95–1.02)		0.98 (0.95–1.02)
Whole pregnancy		1.00 (0.98–1.02)		0.99 (0.96–1.02)*		1.01 (0.98–1.04)
O3 (10 ppb)						
1st trimester		1.01 (0.96–1.06)		1.01 (0.94–1.09)*		0.99 (0.92–1.06)
2nd trimester		0.96 (0.91–1.01)		1.01 (0.94–1.08)*		0.92 (0.85–0.98)*
3rd trimester		0.99 (0.93–1.04)*		0.98 (0.90–1.08)*		0.98 (0.90–1.08)*
Whole pregnancy		0.97 (0.91–1.04)		1.01 (0.92–1.11)*		0.94 (0.85–1.03)*
Abbreviations: CO, carbon monoxide; NO2, nitrogen dioxide; O3, ozone; PM10, particulate mattter ≤ 10 µm in aerodynamic diameter; SO2, sulfur dioxide. aLogistic regression analysis adjusting for sex, maternal age, gestational age, municipal-level socieoeconomic status (SES), season of conception, and year of birth. bLogistic regression analysis adjusting for sex, maternal age, municipal-level SES, season of conception, and year of birth. *Point estimates were similar with those from the original analyses, but some confidence intervals were wider.						
